# Association of diet and headache

**DOI:** 10.1186/s10194-019-1057-1

**Published:** 2019-11-14

**Authors:** Soodeh Razeghi Jahromi, Zeinab Ghorbani, Paolo Martelletti, Christian Lampl, Mansoureh Togha, On behalf of the School of Advanced Studies of the European Headache Federation (EHF-SAS)

**Affiliations:** 1 Department of Clinical Nutrition and Dietetics, Faculty of Nutrition and Food Technology, Shahid Beheshti University of Medical Sciences, Tehran, Iran; 2 Headache Department, Iranian Center of Neurological Research, Neuroscience Institute, Tehran University of Medical Sciences, Tehran, Iran; 3 Cardiovascular Diseases Research Center, Department of Cardiology, Heshmat Hospital, School of Medicine, Guilan University of Medical Sciences, Rasht, Iran; 4 Department of Clinical and Molecular Medicine, Sapienza University of Rome, Rome, Italy; 5 Headache Medical Center, Ordensklinikum Linz Barmherzige Schwestern, Linz, Austria; 6 Headache Department, Neurology Ward, Sina University Hospital, School of Medicine, Tehran University of Medical Sciences, Tehran, Iran

**Keywords:** Migraine, Diet, Nutrition, Fasting, Elimination diet

## Abstract

The global prevalence of migraine as a primary headache has been estimated as 14.4% in both sexes. Migraine headache has been ranked as the highest contributor to disability in under 50 years old population in the world. Extensive research has been conducted in order to clarify the pathological mechanisms of migraine. Although uncertainties remains, it has been indicated that vascular dysfunction, cortical spreading depression (CSD), activation of the trigeminovascular pathway, pro-inflammatory and oxidative state may play a putative role in migraine pain generation. Knowledge about pathophysiological mechanisms of migraine should be integrated into a multimodal treatment approach to increase quality of life in patients. With respect to this, within the integrative health studies growing interest pertains to dietary interventions. Although the number of studies concerning effects of diet on headache/migraine is not yet very large, the current article will review the available evidence in this area. All publications on headache/migraine and dietary interventions up to May 2019 were included in the present review through a PubMed/MEDLINE and ScienceDirect database search. According to the current findings, Ketogenic diet and modified Atkins diet are thought to play a role in neuroprotection, improving mitochondrial function and energy metabolism, compensating serotoninergic dysfunction, decreasing calcitonin gene-related peptide (CGRP) level and suppressing neuro-inflammation. It can also be speculated that prescription of low glycemic diet may be promising in headache/migraine control through attenuating the inflammatory state. Moreover, obesity and headaches including migraine could be attributed to each other through mechanisms like inflammation, and irregular hypothalamic function. Thereby, applying dietary strategies for weight loss may also ameliorate headache/migraine. Another important dietary intervention that might be effective in headache/migraine improvement is related to balance between the intake of essential fatty acids, omega-6 and omega-3 which also affect inflammatory responses, platelet function and regulation of vascular tone. Regarding elimination diets, it appears that targeted these diets in migraine patients with food sensitivities could be effective in headache/migraine prevention. Taken together, dietary approaches that could be considered as effective strategies in headache/migraine prophylaxis include weight loss diets in obese headache patients, ketogenic and low-calorie diets, reducing omega-6 and increasing omega-3 fatty acid intakes.

## Introduction

### Headache epidemiology and etiology

According to the reports of global burden of headache, 2016 [1], The global prevalence of migraine as a primary headache has been estimated as 14.4% in both sexes [1]. Migraine headache has been ranked as the highest contributor to disability in under 50 years old population in the world [2]. Furthermore, it has been evident that women are affected by migraine 2 or 3 times more than men and also experience more disabling, more severe attacks with longer duration and increased risk of recurrent headaches [3]. Based on the number of headache days in a month, migraine is classified into episodic migraine ((EM): having < 15 headache days /month) or chronic migraine ((CM): having ≥15 headache days /month with experiencing migraine features in at least 8 days/month) [4]. Suffering from concurrent disorders such as other neurologic and psychiatric disorders, chronic pain, cardiovascular diseases, gastrointestinal (GI) complaints, allergy or /asthma, and obesity would also make the treatment more complicated. These comorbidities may additionally be involved in the transformation from EM to CM [5, 6]. Irrespective of treatment modalities applied, trigger control, and lifestyle modification are indispensable to the successful management of migraine [7].

Therefore, knowledge about pathophysiological mechanisms of migraine should be integrated into a multimodal treatment approach to increase quality of life in patients. With respect to this, within the integrative health studies growing interest pertains to dietary interventions. Although the number of studies concerning effects of diet on headache/migraine is not yet very large, the current article will review the available evidence in this area. The dietary approaches that will be discussed throughout this manuscript include fasting and carbohydrate restricted diets (ketogenic diet (KD), low-calorie diet, modified Atkins diet (MAD), low glycemic diet (LGD),), weight loss diets, low-fat diet, elimination diet and low sodium diet. Afterwards, the possible mechanisms underlying each diet in protecting against primary headache with a focus on migraine pathogenies will be explored at the end of each section.

### Evidence acquisition

All publications on headache/migraine and dietary interventions up to May 2019 were included in the present narrative review through a PubMed/MEDLINE, Science Direct and Google Scholar database search. The following keywords were used: “diet”, OR “nutrition”, OR “dietary intervention”, OR “ketosis”, OR “fasting”, OR “glycemic index”, OR “carbohydrate”, OR “fat”, OR “protein”, OR “weigh reduction”, OR “obesity”, OR “food elimination”, OR “sodium”, AND “chronic migraine”, OR “episodic migraine”, OR “tension type headache”, OR “headache”, OR “treatment” AND “inflammation”, “endothelial function”, OR “platelet aggregation”, OR “pain”, OR “nociception”. Reference of included articles was evaluated, and relevant studies were also included in the current review. All eligible studies were written in the English language and were performed on adults. A description of the studies on dietary interventions in adults with headache is summarized in Additional file 1 and the studies on pediatric patients and adolescents are summarized in Additional file 2. The majority of included articles were case studies, case-series, case-control and clinical trials.

### Diet and headache

Among lifestyle modalities, nutraceuticals and diet play a notable role in headache/migraine and therefore adjusting one’s diet could be useful in preventing and treating headaches [8–10]. The main components of a comprehensive diet include carbohydrates, proteins, fats, vitamins, and ions. It is not clear whether these dietary factors prevent or provoke headache attacks [11]. The initiation of a headache/migraine attack may occur following consumption of specific food items. These food items should be identified and eliminated [12, 13]. Moreover, making specific dietary recommendations based on patients’ needs, and types of comorbidities could be effective in reducing the frequency of headache or even preventing initiation of an attack [12]. The underlying comorbidities of the patients that have gained a special attention when making dietary advices consist of obesity, seizure, GI disorders, depression and anxiety, and food intolerance [12].

So far, the effects of different types of diets have been studied in relation to migraine and headache [14–18]. It is speculated that dietary interventions could affect headache/ migraine characteristics through a variety of mechanisms. These mechanisms may include affecting serotoninergic dysfunction, neuronal excitability, levels of factors with a role in migraine pathogenesis (such as Calctonin-Gene-Related-Peptide (CGRP), nitric oxide (NO), adiponectin, and leptin), brain mitochondrial function, neuro-inflammation, hypothalamic function, and platelet aggregation [17, 19–30]. For example, obesity, which is highly related to western dietary patterns [31], is also thought to be prevalent among headache patients [32]. It has been proposed that headaches could be improved following reducing excessive weight [32–34].

## Fasting and carbohydrate restricted diets

Before long the great philosophers applied fasting as a means of therapy (https://www.allaboutfasting.com/history-of-fasting.html). KD was initially designed in order to stimulate the ketotic effects of fasting. Using KD for treating refractory epilepsy dates back to the time of Hippocrates [35]. Then after, in recent years it received growing attention as a potential treatment for other neurological disorders [36, 37]. In the following section, the effects of different types of fasting, low caloric, ketogenic, MAD and LGD on headache/migraine will be explored.

In one case report and one case series [38, 39], modified fasting was the mean of producing ketosis in headache patients. In the specific case report, a woman with chronic headache used modified fasting diet with 3–4 high protein/low carbohydrate (200 kcal) shake a day. After ketosis establishment, the headache attacks disappeared. This effect remained for 7 months after stopping fasting [38]. In the mentioned case series study, 51 adults with chronic migraine followed a low-calorie diet (1200–1500 kcal/d) for several months. Significant reduction in headaches days and abortive medication consumption was observed. Twenty eight percent of the sample reached complete remission from migraine headache. Also, continuous improvement was noted for 3 months after stopping low calorie diet [39].

Both KD and the MAD have been widely prescribed in the treatment of patients with intractable epilepsy [15]. Carbohydrate content in KD is highly restricted which results in inducing fasting in addition to rapid weight loss and elevating metabolism of fat and thus producing ketone bodies (KB) [15, 40, 41]. While carbohydrate restriction in the MAD is lesser than KD, MAD does not require a prior induced fasting state, restriction of energy, protein or fluid and is thereby applied on out-patients [40, 42]. Likewise to anticonvulsant medications, the beneficial effects of KD and MAD on the conditions such as neurodegenerative disorders, brain tumors, autism, amyotrophic lateral sclerosis, and migraine have also been of interest as a therapeutic strategy [15, 41, 43]. These type of diets are thought to play a role in neuroprotection, mitochondrial function and enhancement of production of ATP [15, 43].

The application of KD for treating headache dates back to 1928 [44]. In the first case-series, a group of 18 migraineurs treated with KD, half of the studied population reported some relief [44]. Since then up to now, only a few case reports have addressed specifically the encouraging effect of KD on migraine/headache [38, 40, 45, 46]. In a recent study [40], 18 adults suffering from migraine without aura were investigated during interictal state. They were prescribed with KD for 1 month. Significant improvements were reported in frequency and duration of their migraine attacks. This could be explained by the fact that KD regulates the balance between inhibition and excitation at the cortical level as shown by normalizing the neurophysiologic tests findings including interictally decreased visual (VEPs) and median nerve somatosensory (SSEPs) evoked potentials habituation [40].

### Comparison of ketogenic and low-calorie diets

Di Lorenzo and colleagues compared ketogenic and low-calorie (1200–1500 kcal/d) diets, in a group of 108 migraineurs [14]. KD was superior to low-calorie diet with a 90% responder rate, while low calorie diet was not effective [14]. Di Lorenzo et al. [41] have additionally reported migraine remission following ketosis in an open-label study on 96 migraine suffers. During the ketogenic phase (the first month of intervention) in ketogenic very-low-calorie diet or KD group (*n* = 45) a significant improvement in headache related features including frequency of attacks, number of headache days and medications use was demonstrated independent of weight reduction. Continuous improvement was also observed for a couple of months after stopping the diet. It is of note that although the frequency of headache days rapidly decreased in patients who followed KD, it worsened when they stopped the diet throughout the transition period (from first vs. second month of study). Moreover, there was a significant reduction in frequency of headache days and medications use and headache attacks frequency following prescribing an standard low-calorie diet in the other intervention group (*n* = 51) after 3 and 6 months, respectively [41].

### Low glycemic diet (LGD)

Consuming more than half of the total energy (50–55%) from carbohydrate has been generally accepted as a healthy diet for several decades [11]. In some circumstances such as epilepsy, weight management, diabetes, and hyperlipidemia, LGD has been proved as an effective alternative [47]. In LGD, daily carbohydrate intake is restricted to 40–60 g with a glycemic index (GI) of less than 50 relative to glucose [48]. So consumption of white bread, sugar, chocolate, sweets, pastries, rice, potato, corn, jams, honey, molasses, ready-made fruit juices, sugary carbohydrate drinks, watermelon, and melon would be limited [49]. LGD is a therapeutic dietary option with remarkable advantages including its increased tolerability and a low incidence of side effects [50]. In LGD carbohydrate is mainly come from legumes, vegetables, fruits, and high fiber cereals [47].

In a RCT by Evcili et al. in 2018 [49], 350 migraineurs were allocated (*n* = 1:1) either to a low GI diet group or to a prophylactic medications group (who received propranolol, flunarazine, amitriptyline). One month after dietary restriction, frequency of attacks were reduced significantly in both groups. After 3 months, headache intensity was also significantly reduced following the low glycemic index diet [49]. According to the results of different studies, LGD, at least in part, imposes its effect by modifying inflammatory responses. In a clinical study designed to evaluate the impacts of a low glycemic (GI 38) legume-enriched (250 g/d) diet compared to a healthy American diet (GI 69), it was shown that soluble TNF-a receptors II and CRP levels were significantly attenuated by adherence to LGD [51]. To summarize, it can be speculated that prescription of LGD may be promising in migraine control, However, more RCTs are required in order to fully elucidate the effects of LGD on migraine characteristics.

### Suggested mechanisms for the effects of ketosis on headache with a focus on migraine pathogenesis

Despite several animal studies have been conducted on the effects of ketosis on different aspects of metabolism [52, 53], the exact pathway by which it may affect on CSD, and trigeminal activation has not yet been clarified. However, several mechanisms have been proposed in the literature [14, 52, 53]. According to in vitro research, it is thought that ketosis may attenuate the severity of migraine headache through compensating serotoninergic dysfunction, inhibition of neuronal excitability, decreasing CGRP synthesis and release, and cortical spreading depression (CSD) and by brain mitochondrial function improvement [14, 52, 53]. Moreover, studies on mouse models indicated that ketosis may prevent neurogenic inflammation [19, 54] which is believed to play an important role in migraine pathogenesis [28]. Also, animal studies revealed that ketosis might increase neuropeptide Y (NPY) and agouti-related protein (AgRP) levels through stimulating ghrelin secretion from the stomach during fasting state. It has been suggested that hypothalamic AgRP and NPY may reduce CGRP level subsequently [55].

## Weight loss strategies

The relationship between primary headaches and obesity was first suggested by Scher and colleagues in 2003 [56]. In a prospective population-based 11 months follow-up, 3% of controls developed chronic daily headache (CDH). Obese subjects (body mass index (BMI) ≥ 30), had a fivefold increase in the relative risk of developing CDH compared to normal weight individuals. Odds of CDH were three times higher in overweight patients (BMI: 25–29) [56]. In this regard, weight reduction is among the suggested interventions for headaches due to idiopathic intracranial hypertension [33]. Although data about the effects of weight loss on primary headache control is limited, the association between migraine and obesity has been a growing field of interest in the recent years. According to an observational study, subjects with obesity would experience more frequent and severe headaches compared to normal-weight individuals [34]. Besides, both abdominal and general obesity have been reported to be independent risk factors for headache development [32]. Studies concerning the effects of weight reduction on migraine applied two approaches including non-surgical modalities, especially dietary intervention, or surgical approaches, particularly bariatric surgery [57–59]. An open-label trial compared a low-calorie diet with KD found that achieving a significant weight loss through each of these interventions could result in a decrease of headache frequency [41]. In a recent trial, bariatric surgery was compared with a multi-intervention approach including low-calorie diet and aerobic exercise program. With the comparable amount of weight loss, bariatric surgery offered a better improvement in headache days and attack duration than non-surgical interventions [58]. Also, 2 observational studies [57, 59] proposed a decrease in migraine intensity, frequency and disability in obese women suffered from migraine after bariatric surgery [57, 59]. Indeed, these studies showed promising results [57–59]. However, a recent small single blind trial examined the effect of low-calorie diet on migraine and found no significant effect [14].

To conclude, although considerable efforts have been made in this field, the results about the direct effects of weight loss dietary intervention on migraine/headache are not conclusive yet. Differences in assessing and modifying life style parameters might influence the results of the studies on the effect of weight loss in controlling headache attacks. In the following part, the proposed mechanisms of the association between excess body weight and migraine will be discussed. Notwithstanding, when it comes to shared pathophysiological pathways, inflammation received most attention.

### Migraine and metabolic syndrome

In addition to obesity, hypertension, dyslipidemia, insulin resistance, and augmented inflammation, that all are believed to be components of metabolic syndrome, tend to be highly prevalent diseases in migraineurs [49, 60]. Recent studies have reported that insulin level may also be higher among migraineurs. About 11.1% of these patients may suffer from IR [61]. It was also noted that IR might correlate with attacks duration in migraine suffers [62]. Migraine and metabolic syndrome are usually comorbid, though no causal relationship has yet been established [60]. Moreover, the association between metabolic syndrome components and migraine characteristics including frequency of headache attacks, severity and duration needs further studies [60]. Although no specific treatment for migraine and concurrent metabolic syndrome has been proposed to date, general recommendations are given on following a weight reduction plan including diet and physical activity, proper sleep duration, and reducing stress levels [60].

### Suggested mechanisms for the association between obesity and headache with a focus on migraine pathogenesis

#### Three dimensional effect of inflammation, headache, and obesity

The hypothetical relationship between obesity and migraine has been linked to an elevated release of pro-inflammatory markers and neuroinflammation that might be principally involved in migraine pain genesis [28]. Among the studied proinflammatory agents, elevated level of C-reactive protein (CRP), which is known as a marker of systemic inflammation, has been reported both in obese individuals and patients with migraine. It seems there could be an epidemiological association between CRP and migraine headache onset [63, 64]. Furthermore, a rise in pro-inflammatory factors, such as interleukin (IL)-1β, IL-6, tumor necrosis factor (TNF)-α and leptin were reported in obese individuals, while anti-inflammatory agents including adiponectin seem to be increased in this population. These events finally lead to a persistent low-grade inflammatory status [20–22]. On the other aspect, IL-1β, IL-6, TNF-α levels have also been shown to be elevated in migraineurs especially during their attack phases [63, 65–69].

In addition, due to the important role of CGRP in migraine pathogenesis, the neuropeptide and its receptors are predominantly important targets for migraine treatment [29]. On the other hand, evidence proposed an increase in plasma CGRP level in adults with obesity, which is also observed in patients with migraine [29, 30, 70, 71]. Moreover, it has been proposed that the administration of CGRP induces the accumulation of fat in obese animal models. Also in murine model, an elevation of CGRP level was reported before obesity onset [70–73]. Substance P (SP) in another factor which is likely to play a role in migraine attack pathogenesis that was also detected in adipose tissue and shares a role in fat accumulation and the start of the inflammatory cascade related to obesity [70].

#### Adipokines and migraine

Further, in recent years, the relationship between adipocytes released factors, known as adipokines (e.g. adiponectin and leptin) and migraine headache has given more insight into the contribution of adipose tissue in the pathophysiology of migraine [23, 24]. Although more studies are required to make a definite conclusion, the current evidence propose that adiponectin concentration might be increased between attack phases whereas it may be decreased during migraine attacks [23, 24]. It has also been mentioned that the level of this factor might be mediated following prophylactic treatment of migraine with topiramate [23]. Thus, it could be hypothesized that chronic rise in adiponectin level might be beneficial in migraine improvement [23]. This issue might be related to the reported correlation of low level of adiponectin with proinflammatory cytokine secretion and platelet aggregation [22, 74]. Adiponectin may block TNF-α and IL-6 production and on the other hand, it could induce IL-10 and IL-1 receptor antagonist (IL-1 RA) formation [74]. Thus, at lower than normal levels, adiponectin might be nociceptive [58]. On the other hand, reduced adiponectin level appears be involved in increasing the risk of developing obesity, atherosclerosis and diabetes [22, 75]. Additionally, increased leptin level is thought to induce secretion of proinflammatory factors that play a role in migraine (IL-6 and TNF- α) and NO, through NFκβ signaling pathway [24, 74]. The current findings reported leptin administration in Wistar rats could diminish the threshold of pain [76]. On the flip side, enhancement of leptin levels following weight reduction has been noted [23]. However, the results of the researches concerning the association between leptin levels and migraine have not been conclusive yet [23]. Nonetheless, it is likely that migraineurs might have lower leptin levels in ictal phases and higher levels of leptin during inter attack periods. Besides, there may be a negative relationship between leptin and pain intensity [23].

#### Effect of irregular hypothalamic function on weight and headache

Some neurotransmitters, such as serotonin (5HT), are responsible for food consumption and body weight regulation which are controlled by hypothalamus and seems to be involved in sense of fullness [25]. On the other hand, the increment in serotonin concentrations in ictal periods in migraine can possibly be attributed to the secretion of serotonin from platelets that induce vasoconstriction of arteries and affects CSD development [26, 27].

Another appetite regulator, which also might be contributed to migraine, is orexin A. An increase in CSF level of orexin has been observed in migraineurs [63]. Orexin A could have antinociceptive characteristic and may probably play a role in compensatory reaction to pain and also contribute to hunger perception [63]. Additionally, orexinergic system dysfunction may be associated with homeostatic pathways which are involved in risk of attack generation, migraine nociception and characteristics, as well as its premonitory stage including appetite alteration [77]. Evidence showed that orexin A administration in murine model stimulates hunger and postpones the sense of fullness [63]. Therefore, applying the medications that can affect the orexinergic system may ameliorate migraine associated gastrointestinal features [77]. However, more studies are needed in order to address the association between orexin A and migraine headache in obese and non-obese individuals and explore the effects of drugs that target this system in migraineurs.

Besides, available data suggest that hypothalamic NPY may contribute to the etiology of weight gain among migraineurs who received prophylactic treatment [77]. For instance, NPY concentrations in plasma of migraine suffers may be elevated following treatment with flunarizine or amitriptyline. It has been also proposed that the weight gain following drug therapy may probably be related to changes in leptin transport system or sensitivity to leptin [77].

## Low-fat diet

Five studies addressed the effect of low-fat diets as means of migraine/headache prophylaxis [78–82]. In 1999, a trial conducted to assess the role of fat-reduced diet for migraine control in 54 adults. Patients were instructed to restrict their fat intake to less than 20 g/d for 12 weeks, after 28 days of run-in period. They reported a notable reduction in headache frequency, intensity, and need for abortive medication [78]. An open-label, randomized cross-over trial investigated the effect of a diet change in comparison with a placebo supplement on migraine patients. For the first 16-week duration, 42 individuals were randomly allocated in intervention group (who were prescribed a low-fat vegan diet for 4 weeks, followed by elimination diet for 4 weeks, followed by reintroduction diet for the last 8 weeks group, *n* = 21) or placebo group (who were supplemented with 10 mcg alpha-linolenic acid + 10 mcg vitamin E as placebo, *n* = 21). Then after a 4-week washout period was considered and the studied subjects in either group crossed over to the other group. A decrease in headache intensity, frequency, and use of abortive medication were observed following the intervention; However, in the mentioned trial, details regarding dietary fat composition were not noted [79]. In another cross-over study on 63 adults with episodic or chronic migraine, low lipid diet (< 20% of total daily energy intake) for 3 months significantly reduced frequency and severity of headache attacks. In this study, participants did not reduce total fat intake to less than 45 g/d and used olive oil as the main source of fat intake [80]. Additionally, based on the theory of the probable effects of different fat types on headache characteristics, a randomized study assessed the effect of omega-3 and omega-6 intake. Fifty-five adults with CM were either reduced omega-6 fats intake or reduced omega-6 fats along with increased omega-three consumption. After 12 weeks, individuals on high omega-3 combined with low omega-6 diet showed a higher headache improvement compared to headache patients on the reduced omega-6 diet [81]. In another randomized double-blind controlled trial on 80 patients with EM, effect of omega-3 supplementation (2500 mg/d) was compared with either nano-curcumin (80 mg/d) or placebo. After 2 months of supplementation, combination of nano-curcumin and ω-3 fatty acids lowered the expression of TNF-α mRNA and serum level of TNF-α. Headache frequency was also reduced in all treatment groups (incl. Nano-curcumin, omega-3, and combination of omega-3/nano-curcumin), with two-fold higher effect in combination group [82].

### Suggested mechanisms for the association between dietary fat and headache with a focus on migraine pathogenesis

#### Fat intake, inflammation, hypercoagulability and hyperaggregability

Amount and type of fat intake affect inflammatory responses [16]. The balance between the omega-6 and omega-3, two main fatty acids that compete with arachidonic acid as eicosanoid biosynthesis precursor, contribute to inflammatory control in response to the environmental metabolic changes. Prostaglandins (PG), which are made from essential fatty acids, take part in platelet function and regulation of vascular tone. PGs also play the principal role in controlling acute and chronic inflammation [16]. PGE1, downstream metabolite of linoleic acid (omega-6), is one of the most potent vasodilators. PGE1 has been shown to cause headache [83]. On the other hand, omega-3 fatty acids (i.e. eicosapentaenoic acid (EPA) and docosahexaenoic acid (DHA)) might probably attenuate platelet aggregation [84] and affect serotonin biosynthesis pathway or the function of 5HT receptors [85].

It is generally believed that high-fat diet elevates plasma LDL-cholesterol and consequently increases platelet agreeability [86]. Studies reported hypercoagulation in serum samples obtained from healthy subjects after a high-fat meal [78, 87]. On the flip side, it has been suggested that migraine attack could be initiated following and condition that causes platelet aggregation, through serotonin secretion and its consequent effects on blood vessels, and NO and PGs production. The secretion of these factors simultaneously may contribute to head pain initiation in migraine [88].

In particular, it is proposed that vulnerability to migraine is likely to be related to constant low concentration of serum serotonin and increased sensitivity to serotonin agonists during attacks, probably due to a defect in serotonin metabolism [26, 27]. In this regard, suppressing platelet aggregation might have therapeutic value in migraine prevention [88]. Therefore, any modalities in dietary fat intake that results in modulating plasma free fatty acids and plasma lipid profile and consequently reducing platelet aggregation, seems to decrease the frequency and duration of migraine headache [78, 88].

## Elimination diet

Each headache patients may have a specific trigger or a unique set of triggers. It is known that certain types of foods and beverages can act as headache triggers [13]. Cheese, chocolate, citrus fruit, alcohol, coffee, tomatoes, carbohydrates, leavened products and red wine are among the proposed foods that may trigger migraine attacks [13, 89, 90]. However, there is not any consensus between previous studies on identifying food triggers in headache. For example, as mentioned, chocolate have been introduced as one of triggering foods of headache; while a double-blind trial by Marcus et al. [91] performed in order to assess the effects of chocolate compared to carob on 63 female subjects suffering from with chronic headache, yielded different results. The trial was conducted following prescribing a diet in which vasoactive amine-rich foods were restricted for 2 weeks. However, after administration of chocolate and carob (both two samples), there was not any differences in provocative effects of these agents on headache [91].

Moreover, there has been speculation about the way that food triggers may act in migraine attack initiation and some probable mechanisms are proposed: the ‘amine hypothesis’, “allergic” mechanism or histamine/NO caused vasodilatation; though, none of these suggested mechanisms has yet been established by adequate evidence [13].

In the following paragraphs, the studies regarding elimination diets in headache patients have been described. A few studies assessed the effect of elimination diets in controlling headache among adults. Two randomized controlled trials (RCT), applied the personalized method for eliminating trigger food in migraine suffers, using IgG antibodies to food antigens [92, 93]. Although, the 12-week parallel-group trial on patients with migraine like headaches that examined the impact of the elimination diet compared to a sham diet failed to show any differences between the 2 studied arms [93], the other study demonstrated beneficial effects in reducing migraine headaches [92]. In this research, the effect of the eliminating diet in migraineurs, who also suffered from irritable bowel syndrome was explored. It was reported that a diet excluding provocative foods in comparison with provocation diet could effectively attenuate the symptoms of both disorders with positive effects on patients quality of life [92]. Similarly, a small cross-over RCT showed that individualized elimination diet could reduce migraine frequency and abortive medication need, compared to a standard diet after six-week [18].

Some headache suffers report that specific foods only provoke headache in combination with stress or extended physical exercise. Both conditions are identified as headache triggers and lead to the production of proinflammatory cytokines [18]. Therefore, it seems that applying elimination diets in headache patients with food sensitivities could be effective in preventing migraine attacks, though more studies are needed [94].

Some patients may also be sensitive to other triggers like histamine, which is related to impairment of detoxification caused by low activity of di-amino-oxidase. Histamine thus, may play an additional role which should be considered in the elimination diets [18]. A sample of 28 patients who suffered from chronic headache attacks were assigned to complete a histamine-free diet for 4 weeks. Nineteen patients had a 50% or greater decline in their headache attacks. Also, the number of headache attacks and analgesic medication consumption significantly decreased following the histamine-free diet [95].

With respect to the probable link between migraine and allergy [96, 97], and due to the beneficial effects of histamine-reduced diet on histamine intolerance symptoms and allergic disorders [98, 99], it can be hypothesized that this type of diet might be promising in migraine control particularly among allergic patients.

### Suggested mechanisms for the effects of elimination diet on headache with a focus on migraine pathogenesis

The mechanisms of IgG-mediated food allergy have not been entirely clarified, but it has been suggested that an increase in production of pro-inflammatory mediators and IgG antibodies through food allergy reaction can induce an inflammatory state that may play a crucial role in the migraine pathophysiology [92]. In both migraine and food sensitivities, inflammation induced by food could make the pro-inflammatory environment which is needed for the induction of headache by other triggers [18]. In this regard, when we focus on inflammation caused by food, a specific indicator is required. Except for IgG4, all IgG subclasses can cause an inflammatory response in exposure to the respective antigen [18]. Accordingly, specific IgG can thus be considered as an ideal tool for a vast number of foods to identify individually suspected food items and enables adjusting eating habits in order to prevent chronic inflammation and occurrence of migraine in sensitized patients [18].

## Low sodium diet

According to the findings of a large sample population-based cohort study there might be a negative relationship between blood pressure and headache occurrence [100].

Therefore, it may be logical to anticipate that dietary interventions that reduce blood pressure, could also lower headache occurrence. In this regard, certain nutritional strategies for lowering blood pressure including dietary approach to stop hypertension (DASH) diet and controlling the amount of sodium intake [101, 102], could be considered as a matter of interest in studies on headache prophylaxis. Available evidence on sodium intake in relation to headache has mainly focused on monosodium glutamate (MSG) intake on headache initiation [103]. Otherwise, in a descriptive study on 266 women with migraine headache, severe headache (measured by visual analogue scale (VAS): 8–10) was 46% less prevalent in subjects with the greatest adherence to the DASH diet. Also, the frequency of moderate headaches (VAS: 4–7) was 36% lower in this group compared to the individuals with lowest adherence [104]. However, the result of the only multicenter, randomized clinical trial on the effect of DASH and low-sodium diet on headache is to somehow different. The study was performed on 390 participants in three 30-days phases (i.e. 1: high sodium diet, 2: intermediate sodium diet and 3: low sodium diet in a random allocation). The occurrence of headaches was not different in DASH group compared to controls, following either phases of low, intermediate and high sodium diets. However, headache risk was lower in low versus high sodium intake, both in DASH diet and control groups [105]. In sum, according to these findings, the current data on the effects of dietary sodium intakes on headache characteristic is not conclusive yet. Thereby, except for those migraineurs suffering from concurrent hypertension [106], more researches are needed to be able to make certain advice for optimal sodium intake in migraine patients.

### Suggested mechanisms for the effects of low sodium diet on headache

Dietary sodium may aggregate headache attacks via direct influence on increasing blood pressure or through inducing endothelial dysfunction [106]. However, due to lack of well-designed clinical trials, and according to the current findings, there is not a persuasive basis for probable beneficial effects of a low sodium diet on migraine. Thus, in vivo and in vitro animal studies and further well-designed clinical trials are needed to clarify the effects of dietary sodium on migraine/headache pathogenesis.

## Conclusion

According to the present review, different nutritional interventions might be effective in migraine and their associated symptoms. There are different types of diets that are thought to attenuate migraine headache. For example, KD and MAD might play a role in neuroprotection, mitochondrial function and energy metabolism, compensating serotoninergic dysfunction, decreasing CGRP level, suppressing neuro-inflammation and CSD, all may be involved in the pathophysiology of migraine. It can also be speculated that prescription of low glycemic diet may be promising in headache/migraine control through attenuating the inflammatory state. Moreover, obesity and headaches especially migraine could be attributed to each other through mechanisms like inflammation, and irregular hypothalamic function. Thereby, applying dietary strategies for weight loss may also ameliorate headache/migraine. Another important dietary intervention that might be effective in headache/migraine improvement is related to balance between the intake of essential fatty acids, omega-6 and omega-3. These dietary approaches could affect inflammatory responses, platelet function and regulation of vascular tone. Regarding elimination diet, it could mostly be effective in migraine patients with food sensitivities to prevent the headaches.

Because in some sections it was not possible to differentiate headache and migraine in included articles, and given there is a dearth of rigorous RCTs in the field of diet and migraine, the results of present review should be should be completed by the future studies. Taken together, dietary approaches that could be considered as effective strategies in headache/migraine prophylaxis include weight loss diets in obese headache patients, ketogenic and low-calorie diets, reducing omega-6 and increasing omega-3 fatty acid intakes.

## Supplementary information

**Additional file 1:Table S1.** A description of the studies on dietary interventions in adults with headache.

**Additional file 2:Table S2**. A description of the studies on dietary interventions in children and adolescents with headache.

## Data Availability

All included references in the present review article are available on the Internet.

## Abbreviations

5HT: Serotonin
AgRP: Agouti-related protein
BMI: BMI body mass index
CDH: Chronic daily headache
CGRP: Calctonin-Gene-Related-Peptide
CM: Chronic migraine
CRP: C-reactive protein
CSD: Cortical spreading depression
DASH: Dietary approach to stop hypertension
EM: Episodic migraine
GI: Glycemic index
IL: Interleukin
KD: Ketogenic diets
LGD: Low glycemic diet
MAD: Modified Atkins diet
MDA: Malondialdehyde
MSG: Monosodium glutamate
NO: Nitric oxide
NPY: Neuropeptide Y
ORAC: Oxygen radical absorbance capacity
PG: Prostaglandin
PPARα: Peroxisome proliferator-activated receptor
RCT: Randomized controlled trials
ROS: Reactive Oxygen Sepsis
SP: Substance P
TBARS: Thiobarbituric acid reactive substances
TNF-α: Tumor necrosis factor-α
TOS: Total oxidant status
VAS: Visual analogue scale
