# Supplementary material for: Association of diet and headache
Source: J Headache Pain. 2019 Nov 14;20(1):106. doi: 10.1186/s10194-019-1057-1 (PMC6854770; doi:10.1186/s10194-019-1057-1)
Supplement: Supplementary file 1 — Additional file 1:Table S1. A description of the studies on dietary interventions in adults with headache. [file 10194_2019_1057_MOESM1_ESM.docx]

| **Table S2. A description of the studies on dietary interventions in adults with headache.** | | | | | |  |
| --- | --- | --- | --- | --- | --- | --- |
|  | | **Study design** | **Studied population** | Study duration | Results |  |
| **Ketosis generating diets** | Non-randomized prospective open-label study, 1928 (1) | | 18 adults with  migraine | Ketogenic diet | Half of the studied population reported headache improvement over ketogenic diet period |  |
|  | Non-randomized prospective open-label study, 1930 (2) | | 50 adults mainly with severe or refractory migraine | Ketogenic diet for 6 months | 78% of patients benefited from the diet, with 28% achieved complete remission |  |
|  | Non-randomized prospective open-label study, 2017 (3) | | 18 adults with migraine during interictal phase (without aura) | Ketogenic diet for one month | Significant improvement of frequency and duration of migraine attacks were observed.  Ketogenic diet regulated the balance between inhibition and excitation at the cortical level by inducing the normalization of the interictally decreased visual (VEPs) and median nerve somatosensory (SSEPs) evoked potentials habituation (p<0.01) |  |
|  | Non-randomized prospective open-label study, 2015 (4) | | 96 overweight women with migraine. Diet chosen based on patient preference | 1. Ketogenesis diet (for 4 weeks) followed by   transitional diet (n=45) (for 8 weeks), compared to standard low-calorie diet (n=51) (for six months) | In the ketogenic diet group headache features were improved in ketosis period, with continuous improvement up to month six. In low-calorie group, significant improvement was observed in number of headache days and need for abortive medication. |  |
|  | Non-randomized prospective open-label study, 2013 (5) | | 108 adults with  migraine | Ketogenic diet for one month (n=52), compared to standard low calorie diet (n=56) | 90% of patients in the ketogenic diet group had some improvement in migraine frequency and medication use. |  |
|  | Case-report, 2006 (6) | | A woman with chronic headache | Modified fasting for about seven months | After ketosis establishment, she became free of headache. The effects remained seven months after stopping fasting | |
| **Low glycemic diet** | Open-label randomized controlled trial, 2018 (7) | | 350 migraineurs | low glycemic index diet group OR (2) prophylactic medications group receiving propranolol, flunarazine, amitriptyline for 3 months | One month after treatment, frequency of attack was reduced in both groups. After three months, attacks’ intensity decreased in the diet group | |
| **Weight loss** | Case series, 2011 (8) | | 29 obese adults suffering from chronic and episodic migraine | Bariatric surgery with 3 and 6 month follow-up | Post bariatric surgery, headache frequency was reduced by more than 50%. Attack duration, and medication need during attacks were also reduced. Three months post-surgery, chronic migraine was converted to episodic migraine in 5 out of 6 chronic migraineurs. | |
|  | Prospective observational study, 2011 (9) | | 24 adult migraineurs with morbid obesity | Bariatric surgery with 6 months | Number of patients with more than 14 headache days per month was reduced by 16.2%. Also % of patients with 4-12 h headache duration was reduced by 53.2%. MIDAS and HIT-6 score, as well as abortive medication use were decreased. | |
|  | Non-randomized controlled trial, 2018 (10) | | 51 obese women with migraine headache. Bariatric surgery | Bariatric surgery compared to diet/ exercise induced weight loss (n: 25) with 6 months follow up | Headache intensity was significantly reduced one and six months after the intervention in both groups. One month after surgery, 68% became free of headache. Also number of migraine free days was increased by 28.3% in the surgery group and 26.45% in the behavioral therapy group. Significant reduction in attack duration was only observed in the surgery group. The surgery group also showed significant lower intensity and duration of migraine attacks and a significantly higher number of migraine-free days than the diet group in one- and six-month time points. | |
| **Low-fat diet** | Open label trial, 1999 (11) | | 56 adults with migraine | 28-days of run-in period, followed by 28 days of low-fat diet (<20 gr/d), without control group | Fifty-one of the 54 subjects reported a >40% improvement in their headache index, and in 35 of the 54 subjects their headache index improved by 85%-100%. | |
|  | Randomized controlled trial, 2015 (12) | | 55 adults with chronic daily headache | a diet high in omega-3 and low in omega-6 fatty acids (the H3-L6 intervention) compared to a diet low in n-6 fatty acids (the L6 intervention) for 12 weeks | In H3-L6 intervention group, for each SD increase in plasma n-3 DHA derivatives 2-docosahexaenoylglyce, 10% reduction in the number of headache days per month and a 40% reduction in the number of severe headache hours per day was reported.  Also, in H3-L6 intervention group, for each SD increase in plasma Docosahexaenoylethanolamine (DHA-EA), 7% reduction in the number of headache days per month and a 30% reduction in the number of severe headache hours per day (p<0.001) was reported | |
|  | cross-over trial, 2015 (13) | | 63 adults with episodic or chronic migraine | Two months run-in period (habitual diet), Low lipid diet (<20% of total daily energy intake) or normal lipid diet (25-30% of total daily energy intake) for 3 months that crossed- over for the following 3 months | 50% decrease in daily fat consumption (specially saturated fats) plus 20% reduction in total daily energy intake significantly reduced the severity and frequency of migraine attacks even compared to a normal diet with equal amount of energy intake. For example in low fat group the number of attacks reduced from 7.4 ± 7.1 in baseline to 2.8 ± 2.4 at the end of trial. | |
|  | open-label randomized cross-over study, 2014 (14) | | 42 adults with migraine | Intervention group: Low-fat vegan diet for four weeks followed by elimination diet for four weeks followed by reintroduction diet for eight weeks (n: 21)  Placebo group: Placebo(10 mcg linoleic acid and 10mcg vitamin E) once daily for 16 weeks (n: 21) | Significant decrease in weight, number of headaches, severity of worst pain and use of medications over diet period as compared to placebo period | |
|  | randomized double-blind controlled trial, 2017 (15) | | 74 patients with episodic migraine | Four groups:   1. Omega 3 (2500 mg/d) for two months 2. Nano-curcumin (80 mg/d) 3. combination of nano-curcumin/Omega-3 4. Placebo | Headache frequency was reduced in all treatment groups (incl. nano-curcumin, omega-3, and combination of omega-3/nano-curcumin), with two-fold higher effect in the combination group (reduced by −2.09 ± 0.34 attacks/week) | |
| **Elimination diet** | prospective  open-label study 1993  (16) | | 28 adults with chronic  headaches and a history  of food intolerance | Histamine-free diet for  four weeks | 68% of patients had a 50% or greater decline in their headache attacks. Also, the number of headache attacks and analgesic medication consumption significantly decreased following the histamine-free diet | |
|  | cross-over RCT, 2010  (17) | | 35 adults with migraine  without aura | IgG antibodies to food  antigens were measured  and then culprit foods  were (1) eliminated OR (2) continued for  six weeks | Individualized elimination diet could reduce migraine frequency and abortive medication need. | |
|  | single-blind,  parallel-group RCT, 2011  (18) | | 167 participants with self-reported  migraine-like  headaches | IgG antibodies to food  antigens were measured  and then participants were  instructed to remove  culprit foods (n:84) OR (2) matched number of   1. non-culprit foods for 12 weeks (n: 83) | This study failed to show any differences between the 2 studied arms. | |
|  | double-blind, cross-over RCT, 2013 (19) | | 21 patients having migraine and irritable bowel syndrome | Run-in period (usual diet) for 6 weeks. Followed by 6 weeks of  elimination or provocation diet according to Ig-G antibodies production to foods. | A diet excluding provocative foods in comparison with provocation diet could effectively reduce the number, duration and severity of attacks, and also abortive drugs consumption. | |
| **Low sodium diet** | multicenter, randomized clinical trial, 2014 (20) | | 390 participants | DASH diet in three 30-days phases: (1) high sodium diet, (2) intermediate sodium and (3) low sodium in a random allocation compared to  Usual diet in three 30-days phases, namely: (1) high sodium diet, (2) intermediate sodium and (3) low sodium in a random allocation | The occurrence of headaches was not different in DASH group compared to controls, following either phases of low, intermediate and high sodium diets. However, headache risk was lower in low versus high sodium intake, both in DASH diet and control groups | |

References

1. SCHNABEL TG. An experience with a ketogenic dietary in migraine. Annals of Internal Medicine. 1928;2(4):341-7.

2. BARBORKA CJ. Migraine: results of treatment by ketogenic diet in fifty cases. Journal of the American Medical Association. 1930;95(24):1825-8.

3. Di Lorenzo C, Coppola G, Bracaglia M, Di Lenola D, Evangelista M, Sirianni G, et al. Cortical functional correlates of responsiveness to short-lasting preventive intervention with ketogenic diet in migraine: a multimodal evoked potentials study. The journal of headache and pain. 2016;17(1):58.

4. Di Lorenzo C, Coppola G, Sirianni G, Di Lorenzo G, Bracaglia M, Di Lenola D, et al. Migraine improvement during short lasting ketogenesis: a proof‐of‐concept study. European journal of neurology. 2015;22(1):170-7.

5. Di Lorenzo C, Coppola G, Sirianni G, Pierelli F. Short term improvement of migraine headaches during ketogenic diet: a prospective observational study in a dietician clinical setting. The journal of headache and pain. 2013;14(1):P219.

6. Strahlman RS. Can ketosis help migraine sufferers? A case report. Headache: The Journal of Head and Face Pain. 2006;46(1):182-.

7. Evcili G, Utku U, Öğün MN, Özdemir G. Early and long period follow-up results of low glycemic index diet for migraine prophylaxis. Ağrı-The Journal of The Turkish Society of Algology. 2018;30(1):8-11.

8. Novack V, Fuchs L, Lantsberg L, Kama S, Lahoud U, Horev A, et al. Changes in headache frequency in premenopausal obese women with migraine after bariatric surgery: a case series. Cephalalgia. 2011;31(13):1336-42.

9. Bond D, Vithiananthan S, Nash J, Thomas J, Wing R. Improvement of migraine headaches in severely obese patients after bariatric surgery. Neurology. 2011;76(13):1135-8.

10. Jahromi SR, Abolhasani M, Ghorbani Z, Sadre-Jahani S, Alizadeh Z, Talebpour M, et al. Bariatric surgery promising in migraine control: a controlled trial on weight loss and its effect on migraine headache. Obesity surgery. 2018;28(1):87-96.

11. Bic Z, Blix GG, Hopp HP, Leslie FM, Schell MJ. The influence of a low-fat diet on incidence and severity of migraine headaches. Journal of women's health & gender-based medicine. 1999;8(5):623-30.

12. Ramsden CE, Zamora D, Makriyannis A, Wood JT, Mann JD, Faurot KR, et al. Diet-induced changes in n-3-and n-6-derived endocannabinoids and reductions in headache pain and psychological distress. The Journal of Pain. 2015;16(8):707-16.

13. Ferrara L, Pacioni D, Di Fronzo V, Russo B, Speranza E, Carlino V, et al. Low-lipid diet reduces frequency and severity of acute migraine attacks. Nutrition, Metabolism and Cardiovascular Diseases. 2015;25(4):370-5.

14. Bunner AE, Agarwal U, Gonzales JF, Valente F, Barnard ND. Nutrition intervention for migraine: a randomized crossover trial. The journal of headache and pain. 2014;15(1):69.

15. Abdolahi M, Tafakhori A, Togha M, Okhovat AA, Siassi F, Eshraghian MR, et al. The synergistic effects of ω-3 fatty acids and nano-curcumin supplementation on tumor necrosis factor (TNF)-α gene expression and serum level in migraine patients. Immunogenetics. 2017;69(6):371-8.

16. Wantke F, Götz M, Jarisch R. Histamine‐free diet: treatment of choice for histamine‐induced food intolerance and supporting treatment for chronical headaches. Clinical & Experimental Allergy. 1993;23(12):982-5.

17. Alpay K, Ertaş M, Orhan EK, Üstay DK, Lieners C, Baykan B. Diet restriction in migraine, based on IgG against foods: a clinical double-blind, randomised, cross-over trial. Cephalalgia. 2010;30(7):829-37.

18. Mitchell N, Hewitt CE, Jayakody S, Islam M, Adamson J, Watt I, et al. Randomised controlled trial of food elimination diet based on IgG antibodies for the prevention of migraine like headaches. Nutrition journal. 2011;10(1):85.

19. Aydinlar EI, Dikmen PY, Tiftikci A, Saruc M, Aksu M, Gunsoy HG, et al. IgG‐based elimination diet in migraine plus irritable bowel syndrome. Headache: The Journal of Head and Face Pain. 2013;53(3):514-25.

20. Amer M, Woodward M, Appel LJ. Effects of dietary sodium and the DASH diet on the occurrence of headaches: results from randomised multicentre DASH-Sodium clinical trial. BMJ open. 2014;4(12):e006671.
