# Supplementary material for: Association of diet and headache
Source: J Headache Pain. 2019 Nov 14;20(1):106. doi: 10.1186/s10194-019-1057-1 (PMC6854770; doi:10.1186/s10194-019-1057-1)
Supplement: Supplementary file 2 — Additional file 2:Table S2. A description of the studies on dietary interventions in children and adolescents with headache. [file 10194_2019_1057_MOESM2_ESM.docx]

1. **Fasting and Ketogenic diets**

In a non-randomized trial by Farkas et. al. on 18 adolescents suffering from migraine who followed a ketogenic diet for 3 months, it was shown that 38% of the total participants had some relief during diet period (1). Also, in a study by Kassoff et. al. the effects of 12-weeks administration of modified Atkins diet (MAD), was studied on eight adolescents with chronic daily headache. MAD induces ketosis like ketogenic diet, without restriction of energy, fluid and protein. However, the compliance was very poor with only three of the patients completing the study, and frequency of migraine attacks was not reduced in any of the patients (2). However, the compliance was reported to be very poor with only three of the patients completing the study, and frequency of migraine attacks was not reduced in any of the patients (2).

1. **Weight loss diets**

A large retrospective study performed in a number of tertiary-care pediatric headache clinic also indicated a relationship between weight reduction and decrease of headache frequency in overweight children with primary headache (3).

Another study addressed the effect of weight loss via low calorie diet, aerobic exercise and cognitive behavioral training on obese adolescents with migraine for 12 months. They suggested that weight reduction could ameliorate migraine intensity, frequency, and disability (4).

1. **Elimination diet**

In 1983, a non-randomized study explored the impact of an oligoantigenic diet to eliminate different types of common trigger foods in 88 children suffering from migraine headache (5). In 93% of included children in this research, elimination of trigger foods lead to complete or remarkable migraine improvement (5).

In a randomized clinical trial, the effects of a fiber-rich diet along with exclusion of food items high in vasoactive amines on migraine were compared with a fiber-rich diet alone in a sample of 39 children. Although a significant decrease in number of migraine headaches was reported at the end of trial in both groups, no differences were observed in headache characteristics between the two groups (6).

| **Table S1. A description of the studies on dietary interventions in children and adolescents with headache.** | | | | | |
| --- | --- | --- | --- | --- | --- |
|  | | **Study design** | **Studied population** | Study duration | Results |
| **Ketosis generating diets** | Non-randomized prospective open-label study, 2014 (1) | | 18 adolescents with  migraine | Ketogenic diet for 3 months | 38% of the total participants had some relief during diet period |
|  | Non-randomized prospective open-label study, 2010 (2) | | 8 adolescents suffered from chronic daily headache | three months | Frequency of migraine attacks was not decreased in any of the patients. Low compliance rate was observed. |
| **Weight loss** | Retrospective observational study, 2009 (3) | | 913 pediatric patients with different type of headaches who received routine visit plus dietary consultation about health risk of obesity | Routine visit plus dietary consultation about health risk of obesity | In children with overweight, there was a direct correlation between BMI reduction and reduction in headache frequency (r=0.32) |
|  | Prospective open-label study, 2013 (4) | | 135 adolescents with migraine | Low-calorie diet, exercise program and cognitive- behavioral consultation for 12 months | Headache intensity, frequency, disability, and use of abortive medication, were decreased after 6 and 12 months of study. |
| **Elimination diet** | Non-randomized trial followed by a double-blind cross-over period, 1983 (5) | | 99 children with  migraine | Oligoantigenic diet with  elimination of multiple  trigger foods in non-individualized  manner with  subsequent double-blind  cross-over period involving  reintroduction of  provocative foods r placebo (patients were received tins of foods which contained provocative food in treatment group) | In 93% of included children in this research, elimination of trigger foods lead to complete or remarkable migraine improvement |
|  | RCT, 1987  (6) | | 39 children with migraine | 1. Fiber-rich diet with   exclusion of foods  with vasoactive amines (n:19) OR (2) Fiber-rich diet without  exclusion of foods with  vasoactive amines  (n: 20) for eight weeks | Although a significant decrease in number of migraine headaches was reported at the end of trial in both groups, no differences were observed in headache characteristics between the two groups. |

**References**

1. Farkas M, Mak E, Richter E, Farkas V, editors. EHMTI-0336. Metabolic diet therapy in the prophylactic treatment of migraine headache in adolescents by using ketogenic diet. The journal of headache and pain; 2014: Springer.

2. Kossoff E, Huffman J, Turner Z, Gladstein J. Use of the modified Atkins diet for adolescents with chronic daily headache. Cephalalgia. 2010;30(8):1014-6.

3. Hershey AD, Powers SW, Nelson TD, Kabbouche MA, Winner P, Yonker M, et al. Obesity in the pediatric headache population: a multicenter study. Headache: The Journal of Head and Face Pain. 2009;49(2):170-7.

4. Verrotti A, Agostinelli S, D'egidio C, Di Fonzo A, Carotenuto M, Parisi P, et al. Impact of a weight loss program on migraine in obese adolescents. European journal of neurology. 2013;20(2):394-7.

5. Egger J, Carter CM, Wilson J, Turner MW, Soothill JF. Is migraine food allergy? A double-blind controlled trial of oligoantigenic diet treatment. Lancet (London, England). 1983;2(8355):865-9.

6. Salfield SA, Wardley BL, Houlsby WT, Turner SL, Spalton AP, Beckles-Wilson NR, et al. Controlled study of exclusion of dietary vasoactive amines in migraine. Arch Dis Child. 1987;62(5):458-60.
